# Sporotrichosis: *In silico* design of new molecular markers for the *Sporothrix* genus

**DOI:** 10.1590/0037-8682-0217-2022

**Published:** 2023-03-06

**Authors:** Blenda Fernandes, Emanoelle Fernandes Rutren La Santrer, Sônia Maria de Figueiredo, Fabiana Rocha-Silva, Cláudia Barbosa Assunção, Amanda Gabrielle Abreu, Iara Furtado Santiago, Susana Johann, Rachel Basques Caligiorne

**Affiliations:** 1 Hospital Santa Casa de Belo Horizonte, Ensino e Pesquisa, Belo Horizonte, MG, Brasil.; 2 Universidade Federal de Ouro Preto, Escola de Nutrição, Programa de Pós-Graduação em Saúde e Nutrição, Ouro Preto, MG, Brasil.; 3 Universidade Federal de Minas Gerais, Instituto de Ciências Biológicas, Departamento de Microbiologia, Belo Horizonte, MG, Brasil.

**Keywords:** *Sporothrix* sp, Polymerase chain reaction, Sporotrichosis, Molecular diagnosis

## Abstract

**Background:**

Sporotrichosis, a cosmopolitan mycosis caused by dimorphic fungi of the *Sporothrix* complex, affects humans and animals. This study aimed to develop new molecular markers for *Sporothrix* genome detection in biological samples using PCR.

**Methods:**

A specific region of DNA sequences from the *Sporothrix* genus, publicly available in GenBank, was chosen for primer design. After testing the *in silico* specificity of these primers, *in vitro* specificity was evaluated using the PCR technique.

**Results:**

Three specific primers with 100% specificity for the *Sporothrix* genus were generated.

**Conclusions:**

PCR using the designed primers can be used to develop molecular diagnostics for sporotrichosis.

Sporotrichosis is a cosmopolitan subcutaneous mycosis that affects humans and animals and is common in tropical and subtropical regions^1^ ^,^ ^2^. The disease is caused by thermally dimorphic fungal complexes of *Sporothrix*, which comprises *S. brasiliensis*, *S. globosa*, *S. schenckii*, *S. luriei*, and *S. mexicana* species. Other known species of this genus that share genetic similarities include *S. pallida*, S*. nivea*, and *S. albican* ^3^. The species *S. brasiliensis* is the most virulent of this genus and is commonly related to zoonotic transmission, whereas *S. schenckii* and *S. globosa* are mostly isolated based on environmental transmission^3^ ^,^ ^4^. 

Mycosis diagnoses are time-consuming. Isolation and visualization of the agent in biological samples are considered the gold standard, but this is challenging^5^ ^,^ ^6^. The preferred agar for fungal culture is Sabouraud dextrose owing to its low cost and its widespread ability to yield growth of most fungi^6^. However, growth can be slow and the development of characteristic structures used to identify these fungi is non-specific. Culture growth can take up to 10 days depending on the clinical sample; isolation and identification can take twice that amount of time. Various serologic methods can be used to assist diagnosis by identifying atypical forms of sporotrichosis or as a method of healing control. Some serologic tests used in this context include the highly specific and sensitive latex agglutination, immunodiffusion, the complement fixation test, ELISA, western blotting, and immunofluorescence^5^.

Biomolecular technology and bioinformatics have become powerful tools facilitating the creation of specific biomarkers for each pathogen^7^. In this context, polymerase chain reaction (PCR) has become a powerful tool to speed up the diagnosis process. Owing to the sensitivity afforded by PCR, diagnosis is possible in the earliest stages of infection, and this was shown to be a reliable strategy for more efficient and refined diagnostics^7^ ^,^ ^8^. Kano *et al.* (2005) reported *Sporothrix* DNA detection and sex-level identification using skin biopsies from cats with sporotrichosis^8^. Successful detection and identification of *Sporothrix* spp. via PCR in cats are relevant because felines are essential transmission vectors for humans. Detection using PCR is thus helpful for the prevention of these infections. 

PCR-based assays are fast, easy to perform, simple to interpret, and highly discriminatory^7^. Nested PCR, for example, is an enhanced PCR reaction that allows for the amplification of products that are otherwise not visualized in a single PCR reaction^9^. In this technique the amplified fragment in the first reaction called “Out” is further amplified to allow fragments to be finally visualized in the second reaction called “In.” Nested PCR consists of two steps, wherein approximately 1 μL of the amplified PCR product from the “Out” step is used for the second reaction “In.” This technique showed a sensitivity of 40 fg of *S. schenckii* DNA, detecting up to only one colony-forming unit in tissue samples^7^ ^,^ ^9^. Isolates from different parts of the world were tested, and the nested PCR showed high sensitivity and specificity. A study by Hu et al., 2003, indicated that nested PCR is 100 times more sensitive than the first PCR, suggesting that it is a highly sensitive and specific method for detecting *S. schenckii* target DNA in biological samples^10^. 

The development of PCR for the diagnosis of sporotrichosis is vital because the gold-standard diagnosis is time-consuming and not entirely reliable. Accordingly, this *in silico* study presents new molecular markers that anneal to the Internal Transcribed sequences (ITS) of the ribosomal DNA (rDNA) domains specific to the *Sporothrix* genus, which can be used to support species identification and disease diagnostics. 

An rDNA sequence from genus *Sporothrix*, deposited in GenBank, a genomic non-redundant database with available data, was selected from https://www.ncbi.nlm.nih.gov/nuccore/KP132788.1**/**. The partial sequence of 18S region, complete ITS1 sequence, complete 5.8S sequence, and partial sequence of 28S region were used to design specific primers. In terms of the sequence, the primers were designed using the Primer-BLAST tool^11^. After primer design, a search was performed to evaluate their specificity for the genus *Sporothrix*, using BLASTn software available at (https://blast.ncbi.nlm.nih.gov). The primers designed were analyzed *in silico* to verify their complementarity with the genomes of other organisms, especially other pathogenic fungi, such as *Aspergillus* spp., *Histoplasma* spp., *Candida* spp., *Coccidioides immitis*, *Blastomyces dermatitidis*, *Cryptococcus* spp., *Paracoccidioides* spp., and species of melanogenic fungi of the family Herpothrichiellaceae. Complementarity with the human genome was also verified, as these markers will be used to detect the fungal agent in human biological samples.

After *in silico* analyses, *in vitro* tests were performed to confirm the specificity of the designed primers. A validation protocol for the PCR reaction using these primers was not carried out in this study. Thus, only eight biological biospsy samples obtained from patients treated at the Santa Casa Hospital of Belo Horizonte, Minas Gerais, Brazil, were analyzed using the nested-PCR technique. The patients presented clinical and laboratory diagnosis for sporotrichosis. The present study was approved by CONEP through the Research Ethics Committee of Santa Casa de Belo Horizonte Hospital - CAAE: 55549216.2.0000.5138. Total DNA was extracted and purified from the biopsy samples of the patients using the PureLink™ Genomic DNA Mini Kit, Invitrogen kit (USA). Thereafter, the whole DNA was quantified via spectrophotometry using a Nanovue Plus (GE Healthcare Life Sciences, Sweden). 

The DNA extracted from the clinical samples was subjected to PCR reaction using the primers designed. The reaction was performed with a final volume of 10 μL in a solution containing PCR 1´ buffer, 3 mM MgCl_2_, 0.2 μM dNTPs, 1.0 U/μL Taq DNA polymerase (all reagents were from Ludwig, Brazil), 0.2 μM of each forward and reverse primer (Invitrogen, USA) and Milli-Q water; 1 μL of extracted DNA (20.0 ng/μL) was used for each reaction. The amplification cycles were as follows: one first step of 95°C for 5 min, followed by 40 cycles of 95°C for 30 s, 60°C for 30 s, and 72°C for 30 s; with a final step of 72°C for 5 min. 

Negative and positive PCR controls were included in each reaction. The positive control was DNA extracted from an international standard culture of *Sporothrix schenkii* ATCC MF765977, a reference for all reactions. Mixtures containing all reaction components were used as the negative control, with sterile ultrapure water in place of the extracted DNA. The final amplification product was analyzed on a 7% polyacrylamide gel stained with silver nitrate. 

For the first reaction (“Out”), the primers SPOR1 (forward) (5′-TCACAACTCCCAACCCTTGC-3′) and SPOR2 (reverse) (5′-GGGAGAACATGCGTTCGGTA-3′) were used. For the second reaction (“In”), the primers SPOR3 (forward) (5′-ATCTCTTGGCTCTGGCATCG-3′) and SPOR2 (reverse) (5′-GGGAGAACATGCGTTCGGTA-3′) were used, using 1 mL of the 1:10-diluted amplicon of the first PCR as the DNA template. Moreover, a PCR with several fungal species was performed to evaluate whether the designed primers would anneal only with the *Sporothrix* genome using the *S. schenckii* standard sample (ATCC MF765977) as the positive control. The cultures used were as follows: *Aspergillus flavus* URM7326; *Aspergillus terreus* URM 7309; *Aspergillus fumigatus* URM 7315; *Candida albicans* (ATCC 52A); *Candida albicans* (ATCC 18804); *Exophiala dermatitidis* URM 7460; *Criptococus gattii* URM7359; *Fonsecaea pedrosoi* URM3161; *Paracoccidioides brasiliensis* (Pb18); *Paracoccidioidomycosis brasiliensis* Pb01. In addition, a human DNA sample known to be negative for sporotrichosis was used. DNA was extracted from the cultures using the PureLink™ Genomic Plant DNA Purification Kit (Invitrogen™). To control for the genomic DNA quality of these cultures, samples were amplified using additional primers (ITS1, 5′-TCCGTAGGTGAACCTGCGG-3′, and ITS4, 5′-TCCTCCGCTTATTGATATGC-3′)^12^. 

The ITS-18S rDNA was chosen as the genomic region to develop the primers based on a literature search. The “International Society of Human and Animal Mycology (ISHAM) -ITS, reference DNA barcoding database-, the quality standardized tool for routine identification of human and animal pathogenic fungi” was used as the basis to select the coding sequence of the ITS rDNA gene of the genus *Sporothrix* ^12^. BLAST analysis of the primer SPOR2 showed that it aligns only with the *Sporothrix* genus. This primer was used for both reactions of the nested PCR.

The PCR using primers designed specifically for the *Sporothrix* genus and the clinical samples, as well as the positive control, yielded bands of approximately 450 bp in the first “Out” PCR and of approximately 250 bp in the second “In” PCR, as predicted *in silico*. There was no amplification of the negative control (Figure 1). The designed primers showed specificity for the samples tested, namely *S. schenkii and S. brasiliensis*, with no amplification of bands when DNA samples from other species of fungi were tested (Figure 2).

**FIGURE 1:**
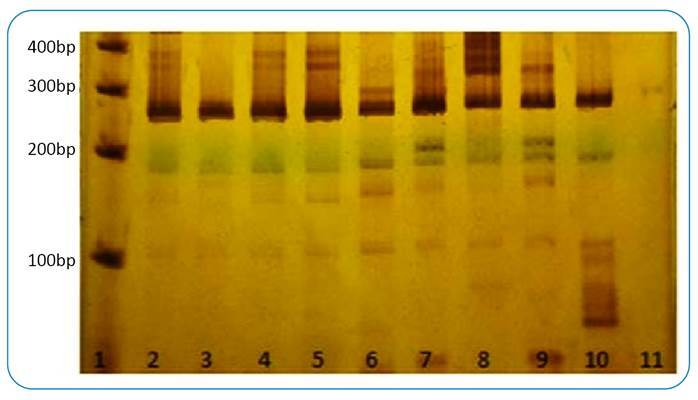
A 7% polyacrylamide gel stained with 2% silver nitrate showing the nested-PCR (“In”) amplification using the primer pair SPOR3 (forward) (5′-ATCTCTTGGCTCTGGCATCG-3′) and R4 (reverse) (5′-GGGAGAACATGCGTTCGGTA-3′), with 1 µL of the amplicon of the first PCR (“Out”), diluted to 1/10, as a DNA template. The fragment amplified was approximately 260 bp, as observed via computational tool analysis. **Lane 1:** molecular weight (MW) standard of 100 base pairs (bp); **lane 2:** DNA extracted from an international standard sample of *Sporothrix schenkii* ATCC MF765977 as a positive control; lanes **3-10:** DNA extracted from lesion biopsies of patients with clinically and laboratory-confirmed sporotrichosis, at Santa Casa Hospital; lane **11:** negative control (CN).

**FIGURE 2:**
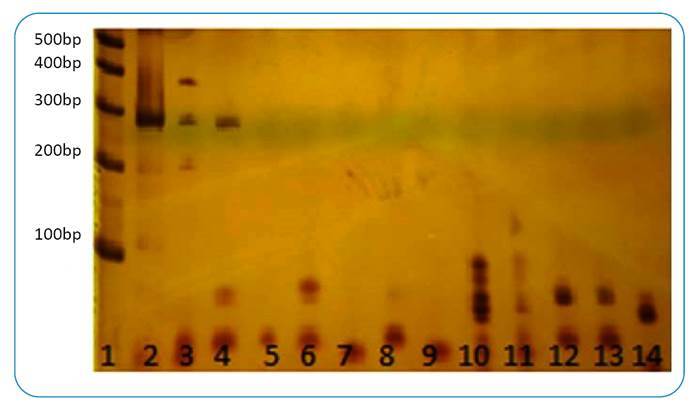
A 7% polyacrylamide gel stained with 2% silver nitrate showing the nested-PCR (In) amplification using the primer pair SPOR3 (forward) (5′-ATCTCTTGGCTCTGGCATCG-3′) and R4 (reverse) (5′-GGGAGAACATGCGTTCGGTA-3′), with 1 ?#61549;L of the amplicon of the first PCR (“Out”), diluted to 1/10, as the DNA template. The fragment amplified was approximately 260 bp, as observed via computational tool analysis. For the PCR specificity test, strains of different fungal species were analyzed. **Lane 1:** molecular weight standard, 100 bp ladder; **lane 2:** DNA extracted from an international standard sample of *Sporothrix schenkii* ATCC MF765977 as a positive control for the reaction; **lanes 3-4:** DNA extracted from biopsies of patients presenting with sporotrichosis confirmed by culture, showing bands of approximately 260 bp; **lane 5:** DNA extracted from international standard samples of *Aspergillus flavus* URM7326; **lane 6:** *Aspergillus terreus* URM 7309; **lane 7:** *Aspergillus fumigatus* URM 7315; **lane 8:** *Candida albicans* (ATCC 52A); **lane 9:** *Candida albicans* (ATCC 18804); **lane 10:** *Exophiala dermatitidis* URM 7460; **lane 11:** *Cryptococcus gattii* URM7359; **lane 12:** *Fonsecaea pedrosoi* URM3161; **lane 13:** *Paracoccidioides brasiliensis* (Pb18); **lane 14:** *Paracoccidioides brasiliensis* Pb01.

The design of new primers underlies the innovation of this study, as a new molecular marker is being proposed for the detection of the *Sporothrix* genome in biological samples. These primers anneal to a specific region of the *Sporothrix* complex genome, allowing them to be used as an efficient diagnostic marker. The target sequence was chosen because it is a vast region of ITS rDNA (partial 18S, complete ITS1, 5.8S complete, and partial 28S)^12^. Statistical analysis of sequences deposited in GenBank showed that this sequence aligns with sequences from several strains of the *Sporothrix* genus, including the most common species in Brazil, *S. schenckii*, *S. globosa*, and *S. brasiliensis*, as demonstrated in a study by Zhou et al., 2014^13^. BLAST*n* analysis showed 100% identity between the target sequence and the sequence from genus *Sporothrix,* thus confirming that the sequence chosen is reliable for designing specific primers.

BLAST*n* analysis of the primer SPOR1 showed that it aligns only with the *Sporothrix* genus and *Ophiostoma*, considering samples deposited in GenBank to the present day. These species, belonging to the Ophiostomatales order, have been described in previous studies related to *Sporothrix* ^10^ ^,^ ^14^. Thus, alignment with *Ophiostoma* sequences does not affect the specificity of the designed primers as *Ophiostoma* is not of clinical importance. This fungus is isolated from trees with no reports of causing human infection. 

BLAST*n* analysis of the SPOR3 primer demonstrated that it aligns with sequences of several fungal species. Therefore, it should only be used for the “In” reaction of the nested-PCR, so that it will only anneal with the fragment amplified by the first “Out” reaction of nested PCR. Nested PCR is used to amplify products that would not be visualized after only one PCR reaction. Thus, it allows for the visualization of fragments based on more pronounced bands associated with a second reaction, as demonstrated in different studies^8^ ^,^ ^9^ ^,^ ^10^ ^,^ ^12^.

BLAST*n* analysis of SPOR2 (reverse) primer resulted in 100% specificity. Considering the samples deposited in GenBank until the present time, SPOR2 aligned only with sequences from *Sporothrix* spp. This primer was used in the “Out” and “In” reactions of the nested PCR. Thus, the *in silico* analyses demonstrated that these primers are specific and of good quality; hence, they can be used to detect *Sporothrix* genomes for the diagnosis of sporotrichosis.

Results of the PCR “Out” reaction showed that the tested samples and positive control yielded bands between 400 and 500 base pairs, which corroborates the results from *in silico* analyses suggesting a fragment of 449 base pairs. The PCR “In” results showed that samples tested, and the positive control yielded bands between 200 and 300 base pairs, which corroborates the results from *in silico* analyses that suggested a fragment of 260 base pairs. PCR using the designed primers showed 100% specificity, in line with the results from a previous study by Rodriguez et al., 2014^15^. This study indicated that PCR can be a highly specific method for detecting *S. schenckii* target DNA in biological samples^9^.

These studies indicate that PCR can be a highly sensitive method for detecting *Sporothrix* target DNA in biological samples, and it can also be used for healing control as it can detect even minimal amounts of DNA in samples. Based on the results showing high specificity among the species compared (100%) and high DNA detection rate, the sequences of the primers SPOR1, SPOR2, and SPOR3 were registered in the National Institute of Property (INPI), under the number BR 10 2017 028202 3. As a final note, this study provides perspectives to continue developing molecular diagnostic methodologies for sporotrichosis and other systemic mycoses as PCR can be applied to a broad spectrum of infectious diseases.
